# A prospective cohort study comparing household contact and water *Vibrio cholerae* isolates in households of cholera patients in rural Bangladesh

**DOI:** 10.1371/journal.pntd.0006641

**Published:** 2018-07-27

**Authors:** Christine Marie George, Khaled Hasan, Shirajum Monira, Zillur Rahman, K. M. Saif-Ur-Rahman, Mahamud-ur Rashid, Fatema Zohura, Tahmina Parvin, Md. Sazzadul Islam Bhuyian, Md. Toslim Mahmud, Shan Li, Jamie Perin, Camille Morgan, Munshi Mustafiz, R. Bradley Sack, David A. Sack, O. Colin Stine, Munirul Alam

**Affiliations:** 1 Department of International Health, Johns Hopkins Bloomberg School of Public Health, Baltimore, Maryland, United States of America; 2 International Centre for Diarrhoeal Disease Research, Bangladesh, Dhaka, Dhaka, Bangladesh; 3 University of Maryland School of Medicine, Baltimore, Maryland, United States of America; Massachusetts General Hospital, UNITED STATES

## Abstract

**Background:**

Household contacts of cholera patients are at a 100 times higher risk of developing cholera than the general population. The objective of this study was to examine the incidence of *V*. *cholerae* infections among household contacts of cholera patients in a rural setting in Bangladesh, to identify risk factors for *V*. *cholerae* infections among this population, and to investigate transmission pathways of *V*. *cholerae* using multilocus variable-number tandem-repeat analysis (MLVA).

**Methodology/Principal findings:**

Stool from household contacts, source water and stored water samples were collected from cholera patient households on Day 1, 3, 5, and 7 after the presentation of the index patient at a health facility. Two hundred thirty clinical and water *V. cholerae* isolates were analyzed by MLVA. Thirty seven percent of households had at least one household contact with a *V*. *cholerae* infection. Thirteen percent of households had *V*. *cholerae* in their water source, and 27% had *V*. *cholerae* in stored household drinking water. Household contacts with *V*. *cholerae* in their water source had a significantly higher odds of symptomatic cholera (Odds Ratio (OR): 5.49, 95% Confidence Interval (CI): 1.07, 28.08). Contacts consuming street vended food had a significantly higher odds of a *V*. *cholerae* infection (OR: 9.45, 95% CI: 2.14, 41.72). Older age was significantly associated with a lower odds of a *V*. *cholerae* infection (OR: 0.96, 95% CI: 0.93, 0.99). Households with both water and clinical *V*. *cholerae*-positive samples all had isolates that were closely related by MLVA.

**Conclusions/Significance:**

These findings emphasize the need for interventions targeting water treatment and food hygiene to reduce *V*. *cholerae* infections.

## Author summary

Household members of cholera patients are at a 100 times higher risk of developing cholera infections than the general population. This risk is highest during the seven days after the cholera patient presents at a health facility. In this study we investigated the rate of cholera transmission within cholera patient households, identified risk factors for household cholera transmission, and performed genetic characterization of cholera strains collected. Stool was collected from patients, their household members, and from water sources and stored water during the seven days after the cholera patient presented at the health facility. A total of 230 human and water *V*. *cholerae* strains were collected and analyzed. Thirty seven percent of households had at least one household member with a *V*. *cholerae* infection. Thirteen percent of households had *V*. *cholerae* in their water source, and 27% had *V*. *cholerae* in stored drinking water. A water source with *V*. *cholerae*, consuming street vended food, and younger age were risk factors for cholera infections for household members of cholera patients. All strains from within households with water and human samples were closely related. These results demonstrate the importance of interventions focusing on water treatment and food hygiene for prevention of cholera.

## Introduction

The World Health Organization estimates that there are 95,000 cholera deaths per year with 2.9 million cases worldwide [1]. Studies have identified risk factors for becoming infected with cholera such as age [2], drinking street-vended water [3], placing ones hands into stored household water [4], bathing in a river [4, 5], eating leftover food [6], eating food prepared by a recently ill food handler [7], not washing hands with soap before eating food [8], and being a first degree relative of a cholera case [9]. These findings indicate that water and food borne contamination are the main transmission routes for *V*. *cholerae* infection.

Previous studies in Bangladesh have demonstrated that household contacts of cholera patients are at a much higher risk of developing a *V*. *cholerae* infection than the general population [2, 10, 11]. The average rates of cholera in Bangladesh are 1.6 cases per 1000 individuals [1], while two studies in rural Matlab, Bangladesh found 240 *V*. *cholerae* infected individuals per 1000 household contacts of cholera cases [1, 5, 10]. Most recently, a study in urban Dhaka, Bangladesh, found 210 *V*. *cholerae* infected individuals per 1000 household contacts of cholera cases [2]. The highest risk for *V*. *cholerae* infections among household contacts is within 7 days of the onset of symptoms in the index case [2, 5, 12]. However despite this high risk, there has been little work done to determine the main transmission routes of *V*. *cholerae* infection for this population. This is likely because it is difficult to elucidate whether cholera transmission to household contacts is from an external source that is shared by household members such as a piped water supply, or by a cholera case infecting family members by contaminating household food or water.

Multilocus variable-number tandem-repeat analysis (MLVA) is a method to distinguish between different strains of *V*. *cholerae* that are typically indistinguishable by methods such as pulse field gel electrophoresis (PFGE) and multilocus sequence typing (MLST) [13–15]. MLVA records the number of repeating sequences found in short DNA fragments at five loci in the genome. Different isolates vary in the number of tandem repeats at each locus thus providing a fingerprint to differentiate between isolates [16]. Our recent study found that *V*. *cholerae* isolates with the same MLVA genotype had significantly fewer pairwise differences by whole genome sequencing (WGS) compared to isolates with different MLVA genotypes [17]. This is consistent with findings from Rashid et al. which found that isolates closely related by MLVA had significantly fewer nucleotides differences by WGS than isolates distantly related by MLVA [18].

In our recent study in urban Dhaka, Bangladesh which followed cholera patient households during the one week high risk period for *V*. *cholerae* infections after the presentation of the index case, we found that stored household drinking water with *V*. *cholerae* and a median free available chlorine concentration below 0.5 mg/L were associated with *V*. *cholerae* infections among household contacts of cholera patients [19]. MLVA and WGS were performed to investigate cholera transmission patterns. The findings showed a combination of person-to-person and water-to-person cholera transmission with the proportions of the two modes varying within and between outbreaks [17].

Building on this previous urban work, our current study focuses on cholera patient households in a rural setting in Bangladesh. Our objective was to examine the incidence of *V*. *cholerae* infections among household contacts of cholera patients in a rural setting, to identify risk factors and investigate transmission pathways for *V*. *cholerae* infections using MLVA. This approach allows for intervention strategies to be identified that can be used to reduce the incidence of cholera among household contacts of cholera patients.

## Methods

### Ethics statement

Informed consent was obtained from all study participants, and study procedures were approved by the research Ethical Review Committee of the International Centre for Diarrhoeal Disease Research, Bangladesh (icddr,b) and the Johns Hopkins Bloomberg School of Public Health IRB.

### Study recruitment

This prospective cohort study was conducted in rural Bakerganj and Mathbaria upazilas in Barisal district of Bangladesh from April 2015 to June 2016. Suspected cholera patients were defined as patients presenting at the Bakerganj and Mathbaria upazila health complexes with acute watery diarrhea (3 or more loose stools over a 24 period). The stool samples from these patients were screened for the presence of *V*. *cholerae* using the Crystal VC Rapid Dipstick test (Span Diagnostics, Surat, India) [20, 21]. All positive findings by dipstick were confirmed by bacterial culture. Cholera patients were defined as diarrhea patients with a stool bacterial culture result positive for *V*. *cholerae*. Screening and study recruitment at Bakerganj and Mathbaria upazila health complexes occurred Saturday to Thursday each week during the study period. The final sample size was based on the number cholera patients that were recruited between April 2015 to June 2016. A cluster was defined as the index cholera patient and their corresponding household contacts. Household contacts were defined as individuals sharing the same cooking pot as the index cholera patient for the previous three days. To be eligible for the study household contacts had to plan to reside in the same household as the index cholera patient for the next week. Eligible household contacts present in the health facility at the time of patient enrollment were invited to participate, and a household visit was made to recruit household contacts within 36 hours of patient enrollment.

Cholera patient households were visited at Days 1, 3, 5, and 7 (Visits 1–4) after the presentation of the index cholera patient at the health facility for clinical and environmental surveillance. For clinical surveillance, household contacts were asked if they had diarrhea (3 or more loose stools over a 24 hour period) or vomiting in the past 48 hours, and a stool sample was collected from willing household contacts at each household visit to test for the presence of *V*. *cholerae* in stool by bacterial culture. For environmental surveillance, water samples were collected from the household’s water source and stored drinking water in the home at each visit to test for the presence of *V*. *cholerae* by bacterial culture. A spot checks was also conducted to observe if soap was present near the latrine and cooking areas of households (within ten steps) as a proxy measure of handwashing with soap behavior, and to assess if household stored drinking water was completely covered [22]. In addition, a structured questionnaire was administered to obtain information on household and individual characteristics.

### Laboratory methods

Stool samples were collected in stool cups from cholera patients and household contacts and water samples were collected in 500 mL bottles. Fecal specimens were enriched in alkaline peptone water (APW) broth for six hours, streaked on Thiosulphate Citrate Bile Sucrose Agar (TCBS) and Taurocholate Tellurite Gelatin Agar (TTGA) plates, and incubated overnight. Serotyping was performed according to previously published methods [23]. Water samples were filtered through 0.22 micron polycarbonate membrane filters and then enriched in APW and cultured as previously described [24]. Two or more colonies were selected from each sample.

MLVA was performed on DNA from 230 *V*. *cholerae* water and clinical isolates from 27 cholera patient households (139 clinical isolates and 91 water isolates). For three households, there was no MLVA data available. DNA was isolated from 5 μl of culture using Prepman (ABI) according to the manufacturer’s instructions. To perform MLVA, the DNA from the *V*. *cholerae* O1 isolates was genotyped at each of five previously identified MLVA loci (VC0147, VC0437, VC1650, VCA0171 & VCA0283) using previously published methods [13]. An MLVA genotype was defined by the alleles at each locus. Genetic relatedness was defined by the similarity of alleles at MLVA loci. If an allele on the large chromosome (VC0147, VC0437, and VC1650) was found to be missing after MLVA analysis, we used the available information from the large chromosome to impute missing values using SAS (version 9.3). If the matching sequences were variable beyond 2%, we assumed that the missing allele could not be deduced and these alleles were not imputed. For locus VC0147, 3 (1% of 230) alleles were imputed, while 99 (43%) and 54 (23%) were imputed for VC0437 and VC1650, respectively. No alleles were imputed for the small chromosome.

### Statistical analysis

Our primary outcomes were: (1) the incidence of cholera infected household contacts defined as an individual with a culture result positive for *V*. *cholerae*, and (2) the incidence of household contacts with symptomatic *V*. *cholerae* infections, defined as a *V*. *cholerae* infection with diarrhea or vomiting. Logistic regression models were performed to estimate the odds of developing a *V*. *cholerae* infection with household and individual level covariates using generalized estimating equations (GEE) to account for clustering within households and approximate the 95% confidence intervals (CI). If there were no *V*. *cholerae* infections in one of the categories, a chi square test was performed.

All analyses were performed using SAS, version 9.4 (SAS Institute Inc., Cary, NC, USA). Pairwise comparisons were made of the number of allele differences in the five locus genotype (e.g. this would be one if a single locus varied) [17]. Fisher’s exact, paired t-tests, and permutation tests were computed using SAS (version 9.3) to analyze MLVA data.

## Results

During April 2015 to June 2016, we screened 1081 diarrhea patients presenting at Mathbaria and Bakerganj health facilities using the Crystal VC rapid dipstick test for *V*. *cholerae*. Fifty-one diarrhea patients had positive results by dipstick, 5 of these individuals refused to participate in our study, and 16 were culture negative for *V*. *cholerae*. All 30 dipstick positive and culture confirmed cholera patients were enrolled in our cohort study. Seventy-six household contacts from these 30 cholera households patients were enrolled (S1 Dataset). Twenty-three households were from Bakerganj and 7 households were from Mathbaria. We observed 3 cholera outbreaks: Outbreak 1 (April -June 2015); Outbreak 2 (October 2015), and in Outbreak 3 (May-June 2016). All three outbreaks had households from both Mathbaria and Bakerganj. Forty-seven percent of index cholera patients (14) and 64% (49) of household contacts were female (Table 1). The mean age was 26 years for index cholera patients and 22 years for household contacts. The mean number of individuals in the household was 5. Eighty-seven percent of households (26) did not completely cover their stored drinking water during the surveillance period (assessed by spot checks), and only 10% (3) reported boiling their household drinking water during the surveillance period. Seventy-nine percent (23) of households had no soap present in the latrine area during the surveillance period and 97% (28) had no soap present in the kitchen area (assessed by spot checks). Twenty-seven percent of households (8) had unimproved latrines using the World Health Organization/ United Nations Children's Fund Joint Monitoring Programme definition [25]. Unimproved sanitation options include pit latrines without a slab or platform, hanging latrines, bucket latrines, and flying toilets. Improved sanitation options include ventilated improved pit latrines, pit latrines with slabs, composting toilets, and flush/pour flush latrines/toilets to piped sewer systems, or septic tanks. Ninety-seven percent of households reported that groundwater was their primary drinking water source, and one household reported pond water and was using a pond sand filter. Seventy-eight percent (59) of household contacts reported consuming water outside the household and 87% (66) reported consuming food outside the household during the surveillance period. Forty-seven percent (36) of household contacts reported eating street vended food during the surveillance period.

**Table 1:** Household and individual level characteristics on visits 1–4, rural Bangladesh.

**Household Level Characteristics**	**% (N)**	**Total Households**
Female Index Cholera Patient	47% (14)	30
Index Cholera Patient Age (Years) (Mean ± Standard Deviation (Min-Max))	26 ± 21 (2–70)	30
Number of Individuals in the Household (Mean ± Standard Deviation (Min-Max))	5 ± 1 (3–8)	30
No Visits with Stored Water Completely Covered during the Surveillance Period	87% (26)	30
No Boiling of Household Water Reported during the Surveillance Period	90% (26)	29
Source Water with Detectable *V*. *cholerae* during the Surveillance Period	13% (4)	30
Stored Water with Detectable *V*. *cholerae* during the Surveillance Period	27% (8)	30
Households with no Soap Observed in the Latrine Area during the Surveillance Period	79% (23)	29
Households with no Soap Observed in the Kitchen Area during the Surveillance Period	97% (28)	29
Unimproved Sanitation Option^1^	27% (8)	30
Household Stores Food Overnight	87% (26)	30
**Individual Level Characteristics**	**% (N)**	**Total Contacts**
Female Contacts	64% (49)	76
Contact Age (Years) (Mean ± Standard Deviation (Min-Max))	22 ± 20 (1–80)	76
Consumed Water Outside of the Household during the Surveillance Period	78% (59)	76
Consumed Food Outside of the Household during the Surveillance Period	87% (66)	76
Consumed Street Vended Food during the Surveillance Period	47% (36)	76

1. World Health Organization/United Nations International Children's Fund Joint Monitoring Program definition includes pit latrines without a slab or platform, hanging latrines, bucket latrines, and flying toilets.

All *V*. *cholerae* strains belonged to serotype Ogawa; and all possessed the cholera toxin gene, *ctxA*. Thirty-seven percent of households (11) had at least one household contact with a *V*. *cholerae* infection, with 13% (4) of households having an infected household contact on the first household visit (Table 2). Twenty percent of households (6) had a household contact with a symptomatic *V*. *cholerae* infection, defined as a *V*. *cholerae* infection accompanied with diarrhea or vomiting in the past 48 hours. Eighteen percent (14) of household contacts had a *V*. *cholerae* infection during the surveillance period and 8% (6) had a symptomatic infection. Five household contacts had a *V*. *cholerae* infection on Visit 1, six on Visit 2, one on Visit 3, and two on Visit 4. Five household contacts had two visits with a stool specimen positive by bacterial culture for *V*. *cholerae*.

**Table 2:** Household and individual level *V*. *cholerae* infection characteristics (visits 1–4), rural Bangladesh.

	**% (N)**	**Total Number**
**Household Level Characteristics**		
Households with ≥1 Contact with an Infection	37% (11)	30
Households with ≥1 Contact with an Infection at Visit 1	14% (4)	28
Households with ≥1 Contact with an Initial Infection detected at Visits other than Visit 1	30% (9)	30
Households with ≥1 Contact with an Symptomatic Infection ‡	20% (6)	30
**Individual Level Characteristics**		
Household Contacts with an Infection	18% (14)	76
Household Contacts with an Infection detected at Visit 1*	7% (5)	72
Household Contacts with an Initial Infection detected on Visits other than Visit 1	12% (9)	76
Household Contacts with Symptomatic Infections‡	8% (6)	76
Household Contact with an Infection by Age Category		
0–5 Years	25% (3)	12
5–14 Years	24% (6)	25
Greater than 14 Years	13% (5)	39
Household Contact with an Infection by Sex		
Male	22% (6)	27
Female	16% (8)	49

‡ Symptomatic infection defined as three or more loose stools over a 24 hour period or vomiting and a positive bacterial culture results for *V*. *cholerae*.

*4 household contacts were not able to provide a stool sample at Visit 1.

Among the 76 household contacts of cholera patients, 28% (21) were the mother of the patient, 24% (18) were a sibling, 12% (9) were the father, 9% (7) were a grandparent, 8% (6) were a spouse, and 20% (15) were another relative. Among those household contacts with a *V*. *cholerae* infection, 4 (29%) were mothers of the index patient, 4 were a sibling (29%), 2 were a spouse (14%), 2 were the father (14%), one was a grandparent (7%), and one was an uncle (7%). During the surveillance period, 15 household contacts reported taking antibiotics, only 5 of these individuals had a symptomatic *V*. *cholerae* infections. One household contact with a symptomatic *V*. *cholerae* infection was referred to a health facility and required IV fluids.

Thirteen percent of households (4) had *V*. *cholerae* in their source water during the surveillance period (all groundwater sources), and 27% (8) had *V*. *cholerae* in stored household drinking water. Seventeen percent (5) of households had *V*. *cholerae* in stored household drinking water on the first visit, and 10% (3) had detectable *V*. *cholerae* in their source water on the first visit. All households with detectable *V*. *cholerae* in their water source had unimproved latrines. All households with *V*. *cholerae* in their source water also had *V*. *cholerae* in stored drinking water. Source water with *V*. *cholerae* only occurred in Outbreak 2. Stored water isolates were from Outbreaks 1 and 2.

Household contacts consuming street vended food had a significantly higher odds of a *V*. *cholerae* infection (Odds Ratio (OR): 9.45, 95% confidence interval (CI): 2.14, 41.72) (*V*. *cholerae* infections: 33% (consumed street vended food) vs. 5% (did not consume street vended food)) (Table 3). Older age in years was significantly associated with a lower odds of a *V*. *cholerae* infection (OR: 0.96, 95% CI: 0.93, 0.99). No household contacts that reported boiling their drinking water during the surveillance period had a *V*. *cholerae* infection compared to 21% of household contacts that did *not* report boiling their drinking water (p = 0.18). Twenty-one percent of household contacts that reported consuming food outside of the home had a *V*. *cholerae* infection compared to no infections for those not consuming food outside the home (p = 0.11). *V*. *cholerae* infections among contacts were not associated with the presence of soap in the kitchen or latrine area.

**Table 3:** Logistic regression models of All *V*. *cholerae* infections^1^ among household contacts of cholera patients on visits 1–4, rural Bangladesh.

	Total Contacts	Odds Ratio^2^	Lower CI	Upper CI	p-value
**Household Characteristics**					
Female Index Cholera Patient	76	0.84	0.22	3.14	0.79
Index Cholera Patient Age (Years)	76	0.99	0.97	1.02	0.71
Number of Individuals in the Household	76	0.93	0.66	1.32	0.69
No Visits with Stored Water Completely Covered during the Surveillance Period	76	2.86	0.47	17.31	0.25
No Boiling of Household Water Reported during the Surveillance Period	76	§	§	§	0.18
Source Water with Detectable *V*. *cholerae* during the Surveillance Period	76	1.56	0.36	6.83	0.56
Stored Water with Detectable *V*. *cholerae* during the Surveillance Period	76	0.52	0.11	2.47	0.41
Households with no Soap Observed in the Latrine Area during the Surveillance Period	74	1.70	0.18	16.00	0.64
Households with no Soap Observed in the Kitchen Area during the Surveillance Period	74	0.49	0.48	1.80	0.83
Unimproved Sanitation Option^3^	76	6.23	0.77	50.47	0.09
Household Stores Food Overnight	76	3.21	0.61	17.05	0.17
**Contact Characteristics**					
Female Contacts	76	0.61	0.19	1.93	0.40
Contact Age (Years)	**76**	**0.96**	**0.93**	**0.99**	**0.011**
Consumed Water Outside of the Household during the Surveillance Period	76	4.61	0.64	33.39	0.13
Consumed Food Outside of the Household during the Surveillance Period	76	§	§	§	0.11
Consumed Street Vended Food during the Surveillance Period	**76**	**9.45**	**2.14**	**41.72**	**0.003**

1. Both symptomatic and asymptomatic infections where a symptomatic infection is defined as a *V*. *cholerae* infection accompanied with three or more loose stools over a 24 hour period or vomiting.

2. Generalized Estimating Equations were used to account for clustering within a household.

3. WHO/UNICEF Joint Monitoring Program definition of unimproved sanitation options which includes pit latrines without a slab or platform, hanging latrines, bucket latrines, and flying toilets.

§ No *V*. *cholerae* infections in one of the categories, therefore confidence intervals could not be calculated.

CI: Confidence Interval.

Contacts with *V*. *cholerae* in their source water had a significantly higher odds of a symptomatic *V*. *cholerae* infection (OR: 5.49, 95% CI: 1.07, 28.08) (25% (*V*. *cholerae* in water source) vs. 6% (no *V*. *cholerae* in water source)) (Table 4). Nine percent of contacts residing in households with no soap in the kitchen area had a symptomatic *V*. *cholerae* infection compared to no *V*. *cholerae* infections among household contacts with soap present in the kitchen area (p = 0.49). All symptomatic *V*. *cholerae* infections occurred among contacts residing in households with unimproved sanitation options (p = 0.12).

**Table 4:** Logistic regression models of symptomatic *V*. *cholerae*^1^ infections among Household Contacts of Cholera Patients on Visits 1–4, Rural Bangladesh.

	Total Contacts	Odds Ratio^2^	Lower CI	Upper CI	p-value		
**Household Characteristics**							
Female Index Cholera Patient	76	0.77	0.14	4.26		0.76	
Index Cholera Patient Age (Years)	76	1.00	0.96	1.04		0.96	
Number of Individuals in the Household	76	0.91	0.56	1.48		0.69	
No Visits with Stored Water Completely Covered during the Surveillance Period	76	0.87	0.13	5.79		0.89	
No Boiling of Household Water Reported the during Surveillance Period	76	§	§	§		0.41	
Source Water with Detectable *V*. *cholerae* during the Surveillance Period	76	**5.49**	**1.07**	**28.08**		**0.04**	
Stored Water with Detectable *V*. *cholerae* during the Surveillance Period	76	1.87	0.34	10.29		0.47	
Households with no Soap Observed in the Latrine Area during the Surveillance Period	74	1.44	0.16	13.09		0.75	
Households with no Soap Observed in the Kitchen Area during the Surveillance Period	74	§	§	§		0.49	
Unimproved Sanitation Option^3^	76	§	§	§		0.12	
Household Stores Food Overnight	76	§	§	§		0.25	
**Household Contact Characteristics**							
Female Contacts	76	0.25	0.04	1.44		0.12	
Contact Age (Years)	76	0.96	0.91	1.01		0.12	
Consumed Water from Outside Household the during Surveillance Period	76	1.41	0.15	13.10		0.76	
Consumed Food Outside of the Household during the Surveillance Period	76	§	§	§		0.32	
Consumed Street Vended Food during the Surveillance Period	76	6.23	0.70	55.48		0.10	

1. A symptomatic *V*. *cholerae* infection is defined as a *V*. *cholerae* infection accompanied with three or more loose stools over a 24 hour period or vomiting.

2. Generalized Estimating Equations were used to account for clustering within a household.

3. WHO/UNICEF Joint Monitoring Program definition of unimproved sanitation options which includes pit latrines without a slab or platform, hanging latrines, bucket latrines, and flying toilets.

§ No *V*. *cholerae* infections in one of the categories, therefore confidence intervals could not be calculated.

CI: Confidence Interval

A total of 230 clinical and water *V. cholerae* isolates were compared by MLVA: 139 clinical isolates from 40 stool samples from cholera patients and their household contacts; 31 source water isolates from 7 source water samples; and 60 stored water isolates from 11 stored water samples (S2 Dataset). These isolates were collected from 27 households across 3 outbreaks: 16 Households in Outbreak 1; 9 Households in Outbreak 2, and 2 Households in Outbreak 3. We identified 31 MLVA genotypes: 7 MLVA genotypes from both clinical and water isolates, 9 genotypes from only water isolates, and 15 genotypes from only clinical isolates. There were 3 alleles at VC0147, 3 at VC0437, 4 at VC1650, 6 at VCA0171, and 6 at VCA0283. There were multiple MLVA genotypes among isolates collected from a single sample. This was similar for clinical samples (mean: 1.9 MLVA genotypes, range: 1–3) and water samples (mean: 2.1 genotypes, range: 1–4), p = 0.53. Eighty one percent of clinical samples (25/31) had at least two isolates with different MLVA genotypes compared to 83% (15/18) of water samples.

Eight households had both clinical and water isolates. When the relatedness of water and clinical isolates from the same household was compared, all households had at least one clinical and water isolate with an identical MLVA genotype or a single locus variant of the same MLVA genotype. However only one household had all clinical and water isolates with identical MLVA genotypes or single locus variants of the same MLVA genotype. For the four households with a positive source water and stored water sample, all had source water and stored water samples with identical MLVA genotypes or single locus variants of the same MLVA genotype. Among the ten households with multiple infected household members, nine out of ten had isolates from different household members with identical MLVA genotypes or single locus variants of the same MLVA genotype. Five out of ten of these households had all clinical isolates with identical MLVA genotypes or single locus variants of the same MLVA genotype.

Isolates collected from the same household had significantly fewer pairwise differences in MLVA loci than those from different households (mean: 0.98 pairwise differences in MLVA loci (same household) vs. 1.79 (different household), p<0.0001). Isolates from the same outbreak also had significantly fewer pairwise differences than those collected from different outbreaks (mean pairwise differences: 1.33 (same outbreak) vs. 2.10 (different outbreak), p<0.0001). When comparing clinical and water isolates, the number of pairwise differences were significantly higher for water compared to clinical isolates (mean 1.79 (water isolates) vs. 1.69 (clinical isolates), p<0.0001).

## Discussion

Nearly 40% of cholera patients had a household member with a *V*. *cholerae* infection during the surveillance period, and 18% of household contacts overall were infected. *V*. *cholerae* was detected in both groundwater and stored water in patient households. Significant risk factors for *V*. *cholerae* infections among household contacts of cholera patients were the presence of *V*. *cholerae* in drinking water sources, consuming street vended food, and younger age. The genetic characterization of *V. cholerae* isolates from cholera patient households showed a high diversity of MLVA genotypes within and between clinical and water samples, and all water and clinical samples within the same household had *V. cholerae* isolates that were closely related. These findings emphasize the need for interventions targeting water treatment and food hygiene to reduce *V*. *cholerae* infections among contacts of cholera patients.

Eighteen percent of household contacts of cholera patients had a *V*. *cholerae* infection in our rural setting in Bangladesh. This is similar to previous studies conducted in rural Matlab, Bangladesh which found 23% and 24% of household contacts of cholera patients to be *V. cholerae* infected [5, 10]. Our findings are also similar to the 19% of household contacts infected in our recent urban cohort of cholera patient households in Dhaka, Bangladesh [26].

Household contacts using drinking water sources with *V*. *cholerae* were significantly more likely to have symptomatic *V*. *cholerae* infections, with 13% of tube wells having detectable *V*. *cholerae*. This finding is consistent with Hughes et al. and Spira et al. conducted in rural Bangladesh where households using a water source positive for *V*. *cholerae* were significantly more likely to have *V*. *cholerae* infections [5, 10]. In Hughes et al. 33% of tube well water samples were positive for *V*. *cholerae*, while in Spira et al. no tube wells had detectable *V*. *cholerae* by bacterial culture only surface water samples [5, 10]. In our current study twice as many stored household water samples were positive for *V*. *cholerae* compared to source water samples (27% vs. 13%). This finding suggests high rates of household contamination of stored water. This result is in contrast to Spira et al. which found similar *V*. *cholerae* concentrations in stored and water source samples (23% vs. 26%) [10]. Hughes et al. did not analyze stored water samples for *V*. *cholerae*. However, this previous study did find that water from cooking, bathing, and washing dishes was often contaminated with *V*. *cholerae* and that this contamination was a significant risk factor for *V*. *cholerae* infections in cholera patient households [5]. Future studies should test all water sources utilized for household tasks for *V*. *cholerae*, not only the household primary drinking water source and stored household drinking water.

Only households with unimproved sanitation options had detectable *V*. *cholerae* in their tube wells. This could be because cholera patients and their infected household members were using these unimproved sanitation options which were contaminating nearby tube wells, or alternatively there could be biofilm growing within tube well pipes from stored household water being used to prime wells. In rural Bangladesh tube wells are often located adjacent to household latrines, making these water sources more susceptible to fecal contamination. Consistent with this an intervention trial conducted in the Philippines found that communities with improved sanitation options had significantly fewer symptomatic *V*. *cholerae* infections compared to communities with no improved water or sanitation access [27]. Furthermore, when sanitation facilities were combined with improved drinking water sources reductions in cholera were doubled compared to sanitation alone.

Both our previous urban cohort study and current rural cohort study found drinking water to be a significant risk factor for *V*. *cholerae* infections among household contacts [19]. However, in our urban setting stored water was a significant risk factor for *V*. *cholerae*, while the source water was significant in our rural setting. This was unexpected given that there was a higher proportion of stored water samples with detectable *V*. *cholerae* in our rural compared to urban site (27% vs. 6%). One potential explanation for this is that households with *V*. *cholerae* in source water had a higher overall burden of fecal contamination in their households, likely from unimproved household latrines.

Street vended food was a sigificant risk factor for *V*. *cholerae* infections in our rural cohort. This is consistent with studies from South Africa, Guatemala, Nigeria, and India where street vended food and water were risk factors for *V*. *cholerae* infections. [3, 8, 28, 29] This is likely because of a recently ill food handler contaminating food or water as was found in rural Micronesia. [7] Younger age was also found to be associated with an increased risk of *V*. *cholerae* infections in our study as was previously shown in rural and urban cohort studies of cholera patient households in Bangladesh. [2, 5] In Weil et al. being less than 14 years of age was a risk factor for *V*. *cholerae* infections, while in Hughes et al. children 5–9 years of age were at highest risk. This association is likely because of young children lacking the naturally acquired immunity to cholera found in older individuals previously exposed.

We observed substantial diversity in MLVA genotypes in clinical and water samples. There were significantly more pairwise differences in water samples compared to clinical samples. This is consistent with the findings from our urban cohort study in Dhaka, Bangladesh. [17] While in Rashed et al. there was greater diversity in clinical isolates compared to water isolates in rural Bangladesh.[30] However this was likely attributed to the low number of water isolates collected.

*Vibrio cholerae* isolates from the same household were more closely related than isolates from different households. This finding is consistent with the source of *V*. *cholerae* infections within cholera patient households being either a shared environmental source in the household such as the drinking water source or street vended food, or person-to-person transmission through poor hygiene practices in the home. Consistent with water-to-person transmission, households with both water and clinical *V*. *cholerae*-positive samples all had isolates that were closely related by MLVA. Our findings are consistent with our urban cohort study where isolates from the same household were also more closely related than those from different households, and the majority of households had water and clinical isolates that were closely related [17]. In support of person-to-person transmission or a shared contaminated source in the household, we found that the vast majority (90%) of infected household members had closely related MLVA genotypes. This is consistent with our urban cohort study which found 82% of household member isolates with identical MLVA genotypes [17]. Future studies are needed that perform whole genome sequencing of water and clinical isolates from cholera patient households in this rural setting to further elucidate transmission pathways for *V*. *cholerae* infections, and these studies should include sampling of all water sources used for household tasks.

This study has several strengths. The first is the rural setting, since recent household contact studies in Bangladesh have all been conducted in urban settings [2, 26]. Second, we collected multiple isolates from all samples allowing us to investigate the diversity of MLVA genotypes within samples. Third, we performed intensive clinical and environmental surveillance that included collecting stool specimens from all enrolled household contacts, not only those presenting with diarrhea or vomiting, and included sampling of water sources and stored household drinking water. Fourth, we included both a risk factor analysis and genetic characterization of water and clinical isolates collected from cholera patient households.

Our study also had a few limitations. First, our sample size was small. We had fewer cholera patients than anticipated during our study period. Second, we did not perform whole genome sequencing on collected isolates which would have provided a higher level of resolution to distinguish the genetic relatedness of isolates collected. In our recent cohort study, however, we found that isolates with the same MLVA genotype were also closely related by whole genome sequencing, with significantly less pairwise differences in single nucleotide-variant counts than isolates with different MLVA genotypes [17]. Third, we did not include community control households. This would have allowed us to estimate the odds of *V*. *cholerae* infections for household contacts of cholera patients compared to community control contacts.

*Vibrio cholerae* in drinking water sources, consuming street vended food, and younger age were important risk factors for *V*. *cholerae* infections among household contacts of cholera patients in our rural setting in Bangladesh. Consistent with the findings from our risk factor analysis, the genetic characterization of strains from cholera patient households showed that the majority of water and clinical samples within the same household had isolates with closely related MLVA genotypes. These results highlight the urgent need for water treatment and food hygiene to reduce *V*. *cholerae* infections among highly susceptible household contacts of cholera patients.

## Supporting information

S1 Checklist STROBE checklist. (DOC)

S1 Dataset Epidemiological data from household contacts and household drinking water. (XLSX)

S2 Dataset Multilocus variable-number tandem-repeat analysis (MLVA) data from cholera patient households. (XLSX)
