# Supplementary material for: A prospective cohort study comparing household contact and water Vibrio cholerae isolates in households of cholera patients in rural Bangladesh
Source: PLoS Negl Trop Dis. 2018 Jul 27;12(7):e0006641. doi: 10.1371/journal.pntd.0006641 (PMC6063393; doi:10.1371/journal.pntd.0006641)
Supplement: S1 Checklist — (DOC) [file pntd.0006641.s001.doc]

STROBE Statement—Checklist of items that should be included in reports of ***cohort studies***

|  | Item No | Recommendation |
| --- | --- | --- |
| **Title and abstract** | 1 | Listed in abstract and title |
|  |  |  |
| Introduction | | |
| Background/rationale | 2 | Introduction Paragraphs 1-4 |
| Objectives | 3 | Introduction Paragraph 5 |
| Methods | | |
| Study design | 4 | Methods Paragraph 2-3 |
| Setting | 5 | Methods Paragraph 2-3 |
| Participants | 6 | Methods Paragraph 2-3 |
|  |  | Methods Paragraph 6 |
| Variables | 7 | Methods Paragraph 6 |
| Data sources/ measurement | 8* | Methods Paragraph 6 |
| Bias | 9 | Methods Paragraph 6 |
| Study size | 10 | Results Paragraph 1 |
| Quantitative variables | 11 | Methods Paragraph 6 |
| Statistical methods | 12 | Methods Paragraph 6 |
|  |  |  |
|  |  |  |
|  |  |  |
|  |  |  |
| Results | | |
| Participants | 13* | Results Paragraph 1 |
|  |  |  |
|  |  |  |
| Descriptive data | 14* | Results Paragraph 1 |
|  |  |  |
|  |  |  |
| Outcome data | 15* | Results Paragraph 1-9 |
| Main results | 16 |  |
|  |  |  |
|  |  |  |
| Other analyses | 17 | Results Paragraph 1-9 |
| Discussion | | |
| Key results | 18 | Discussion Paragraph 1 |
| Limitations | 19 | Discussion Paragraph 10 |
| Interpretation | 20 | Discussion Paragraphs 2-9 |
| Generalisability | 21 | Discussion Paragraph 11 |
| Other information | | |
| Funding | 22 | This is in the Financial Disclosure section |
